# L-Fucose promotes enteric nervous system regeneration in type 1 diabetic mice by inhibiting SMAD2 signaling pathway in enteric neural precursor cells

**DOI:** 10.1186/s12964-023-01311-0

**Published:** 2023-10-05

**Authors:** Hailing Yao, Huiying Shi, Chen Jiang, Mengke Fan, Yurui Zhang, Wei Qian, Rong Lin

**Affiliations:** Department of Gastroenterology, Union Hospital, Tongji Medical College, Huazhong University of Science and Technology, Wuhan, 430022 China

**Keywords:** Enteric neural precursor cells (ENPCs), L-Fucose, Diabetes, Enteric nervous system (ENS)

## Abstract

**Background:**

Diabetes can lead to extensive damage to the enteric nervous system (ENS), causing gastrointestinal motility disorders. However, there is currently a lack of effective treatments for diabetes-induced ENS damage. Enteric neural precursor cells (ENPCs) closely regulate the structural and functional integrity of the ENS. L-Fucose, is a dietary sugar that has been showed to effectively ameliorate central nervous system injuries, but its potential for ameliorating ENS damage and the involvement of ENPCs in this process remains uncertain.

**Methods:**

Genetically engineered mice were generated for lineage tracing of ENPCs in vivo. Using diabetic mice in vivo and high glucose-treated primary ENPCs in vitro, the effects of L-Fucose on the injured ENS and ENPCs was evaluated by assessing gastrointestinal motility, ENS structure, and the differentiation of ENPCs. The key signaling pathways in regulating neurogenesis and neural precursor cells properties, transforming growth factor-β (TGF-β) and its downstream signaling pathways were further examined to clarify the potential mechanism of L-Fucose on the injured ENS and ENPCs.

**Results:**

L-Fucose improved gastrointestinal motility in diabetic mice, including increased defecation frequency (*p* < 0.05), reduced total gastrointestinal transmission time (*p* < 0.001) and bead expulsion time (*p* < 0.05), as well as enhanced spontaneous contractility and electric field stimulation-induced contraction response in isolated colonic muscle strips (*p* < 0.001). The decrease in the number of neurons and glial cells in the ENS of diabetic mice were reversed by L-Fucose treatment. More importantly, L-Fucose treatment significantly promoted the proportion of ENPCs differentiated into neurons and glial cells both in vitro and in vivo, accompanied by inhibiting SMAD2 phosphorylation.

**Conclusions:**

L-Fucose could promote neurogenesis and gliogenesis derived from ENPCs by inhibiting the SMAD2 signaling, thus facilitating ENS regeneration and gastrointestinal motility recovery in type 1 diabetic mice.

Video Abstract

**Supplementary Information:**

The online version contains supplementary material available at 10.1186/s12964-023-01311-0.

## Background

Diabetes mellitus (DM) can cause secondary gastrointestinal motility disorders, including esophageal dysmotility, gastroparesis, and intestinal dysfunction (diarrhea, constipation, flatulence, etc.). The enteric nervous system (ENS), is an independent network of neurons and glial cells, and its injury is a crucial causative factor of gastrointestinal motility dysfunction [1–3]. Diabetes-induced ENS damage can affect almost the entire gastrointestinal tract. However, there is currently no effective treatment for diabetes-induced ENS damage.

In the central nervous system (CNS), endogenous neural precursor cells (NPCs)-which reside in niches-are rapidly activate and differentiate into neurons or glial cells in response to injury. NPCs therapy is currently the most promising approach for neural repair [4]. Importantly, recent studies have shown that a subset of NPCs originated from the vagal or sacral neural crest migrates and colonizes in the gastrointestinal tract during the early embryonic periods, referred to as enteric NPCs (ENPCs). These ENPCs subsequently differentiates into enteric neurons and glial cells [5–8]. In adults, ENPCs and their neurogenesis play crucial roles in maintaining the homeostasis of the ENS [9, 10]. Therefore, ENPCs-based therapy is expected to be a promising novel therapeutic strategy for diabetes-induced ENS damage. However, the physiological change in ENPCs in diabetes remain unknown.

Many molecules have been developed to stimulate or enhance the neurogenic potential of NPCs, and have shown therapeutic benefits in patients and animals with neurological diseases [11]. L-Fucose is the main bioactive component of fucoidan and it’s known to have neuroprotective and neurogenic effects on the CNS [12, 13]. Free L-Fucose could be further processed by glycoproteins into fucosylated glycans, which play an essential role in the development of the nervous system and NPCs [14, 15]. Borisova et al. revealed that the addition of L-Fucose to the diet ameliorated behavioral abnormalities in the odor preference test in chronic colitis mice model [16]. Mountford et al. found that L-Fucose is a prominent functional component of neural tissues such as synaptic membranes [13]. Moreover, L-Fucose rejuvenated intestinal stem cell growth and promoted intestinal epithelial repair in a colitis mouse model [17, 18]. However, the effects of L-Fucose on ENPCs and ENS have not been reported.

Transforming growth factor-β (TGF-β) belongs to a group of the cytokine superfamily, and its binding to receptor activates both SMAD and non-SMAD signaling pathways, including the extracellular signal-regulated kinase (ERK), JNK, and p38 mitogen-activated protein kinases pathways [19]. Current evidence indicates that TGF-β signaling plays a role in various aspects of neurogenesis in the CNS [20, 21]. In addition, TGF-β signaling can regulate the self-renewal and stemness of NPCs, promote the sprouting and elongation of neurites, and mediate axonal specification in the developing brain [22–24]. Despite these studies, the role of TGF-β signaling in ENS neurogenesis remains unclear.

Here, by using ENPCs transgenic reporter mice and primary ENPCs cultures, we aimed to investigate the effects of L-Fucose on ENPCs and ENS regeneration in a mouse model of type 1 diabetes mellitus (T1DM) and to clarify the underlying mechanism.

## Methods

### Animals

Nestin-creER^T2^ mice were crossed with R26-e(CAG-RSR-LSL-DTRGFP-WPRE-pA) and Ngfr-e(2 A-DreER^T2^) mice (purchased from Shanghai Model Organisms Center, Inc) to generate Nestin-creER^T2^ × Ngfr-DreER^T2^: DTRGFP triple transgenic mice, which specially labeled newborn Nestin^+^/Ngfr^+^ cells with green fluorescent protein (GFP) after tamoxifen (TAM) induction (Additional file 1: Fig. S1A). Wild-type C57BL/6J mice were purchased from Beijing HFK Bioscience Co. Ldt. Pregnant female mice (C57BL/6J, E15-E18) were used to isolate ENPCs. All animals were fed in a specific pathogen-free grade facility of the Animal Experiment Center of Tongji Medical College, Huazhong University of Science and Technology under standard animal laboratory conditions [temperature (21–25 °C), humidity (50–60%) and photoperiods light–dark cycle (12 h:12 h)]. All animal studies were approved by the Animal Care and Use Committee of Union Hospital at Tongji Medical College, Huazhong University of Science and Technology.

### Protocols for TAM induction

TAM (25 mg/kg; Sigma, T5648) dissolved in corn oil at a concentration of 20 mg/mL and stored at 4 °C. For labeling Nestin^+^/Ngfr^+^ cells with GFP in Nestin-creER^T2^ × Ngfr-DreER^T2^: DTRGFP mice in vivo, 100 µL TAM was intraperitoneally administered for six consecutive days.

### Induction of diabetes and grouping

The mice were randomly divided into four groups: control, control + L-Fucose, diabetes, and diabetes + L-Fucose (simplified as control, Fuc, DM, and DM + Fuc; respectively). To induce T1DM, the mice were fasted overnight with free access to water, and a single intraperitoneal injection of streptozotocin (150 mg/kg; Sigma, S0130) in 0.1 mol/L citrate buffer (pH 4.5). The mice in the control group were administrated an equal volume of citrate buffer. Seven days later, tail vein blood was collected and measured blood glucose levels using a glucometer. Only those mice with a blood glucose level ≥ 16.7 mmol/L were defined as successful diabetic model and used in the following experiments. Eight weeks after the successful establishment of the diabetic model, L-Fucose (250 mg/Kg; Sigma, F2252) dissolved in 100 µL saline, and was administrated to mice by oral gavage for 28 continuous days (Fuc group: average dosage = 200 mg/animal; DM + Fuc group: average dosage = 140 mg/animal). Additionally, mice in the control and DM groups received 0.9% saline via intragastric administration at the same frequency and volume as those in the L-Fucose-treated groups.

### Isolation, culture, and treatment of ENPCs

The entire gut was dissected from embryonic (E15- E18) mice and rinsed with ice-cold Ca^2+^/Mg^2+^-free phosphate-buffered saline (PBS) supplemented with 1% penicillin/streptomycin (P/S; Gibco, 15,140,122). The tissue was finely diced and incubated in dispase II (1 mg/mL; Roche, 4,942,078,001), collagenase IV (300 u/mL; Roche, 11,088,866,001), DNase I (40 u/mL; Roche, 10,104,159,001), 0.1% bovine serum albumin and 1% P/S dissolved in M199 media for 40 min at 37 °C. Thereafter, the cell suspensions were filtered through a sterile 40 µM cell strainer, centrifuged and resuspended in ENPCs medium [Dulbecco’s Modified Eagle Medium/Nutrient Mixture F-12 (DMEM/F12; Procell, PM150323) containing 1% B27 (STEMCELL Technology, 05731), 20 ng/mL fibroblast growth factor (Peprotech, 450 − 33), 20 ng/mL epidermal growth factor (Peprotech, 315-09), and 1% P/S]. ENPCs were cultured in a humidified incubator (37 °C, 5% CO_2_), with 50% of the medium replaced every other day. Neurospheres were passaged by using accutase (STEMCELL Technology, 07920) for 15 min at 37 °C. Single-cell suspensions were plated on poly-L-lysine-coated (0.1 mg/ml, Sigma, P1399) glass coverslips for immunofluorescence staining.

For the in vitro experiments, ENPCs were divided into three groups: control, high glucose, and high glucose + L-Fucose groups. The effective concentration of L-Fucose for ENPCs proliferation was selected in the concentration gradient experiment. ENPCs were maintained in a medium containing either 5- or 30-mM glucose for 72 h, with the addition of L-Fucose (30 mg/mL) to the culture medium for the final 24 h.

### In vivo functional gastrointestinal motility tests

The total gastrointestinal and colonic transit time were measured in mice fasted overnight. 300 µL carbon black suspension (5% powdered carbon suspended in 10% gum arabic) was given to mice by oral gavage. The total gastrointestinal transient time was determined as the first fecal pellet containing the black carbon expulsed. To measure colonic transit time, the mice were lightly anesthetized with isoflurane, and a 3 mm glass bead was inserted into the colon (3 cm from the anus). The latency for bead expulsion was recorded as the colonic transit time. To measure defecation frequency, the mice were fed freely, and feces were collected for 5 h.

### Tissue harvesting

After the completion of gastrointestinal motility testing in vivo, mice were anesthetized with isoflurane. The entire colon was harvested, and different regions were allocated for different analyses. The proximal colon was harvested for western blot analysis, and the middle section of the colon was embedded in paraffin for preparation of transverse sections. The distal colon was removed for myenteric plexus immunofluorescence analysis and smooth muscle activity analysis.

### Intestinal smooth muscle activity recording

The mice were euthanized by cervical dislocation. The colon tissue of each group was removed, opened along the mesentery, then rinsed in oxygenated (95% O_2_ + 5% CO_2_) Kreb’s buffer (NaCl 119 mmol/L, KCl 4.7 mmol/L, NaHCO_3_ 25 mmol/L, NaH_2_PO_4_ 1.2 mmol/L, MgSO_4_ 1.2 mmol/L, glucose 11.1 mmol/L, CaCl_2_ 2.5 mmol/L, pH 7.30–7.40). The mucosa and submucosa were gently removed, and circular smooth muscle strips (1 cm in length and 3 mm in width) of the distal colon were made. These muscle strips were mounted between the L-shaped tissue hook and the isometric force transducer (AD Instruments, Dunedin, New Zealand) and immersed in a tissue bath filled with 25 mL oxygenated Kreb’s solution at 37 ℃. The muscle strips were then allowed to equilibrate for 30 min under a preload of 1 g to achieve stable spontaneous contraction. Spontaneous smooth muscle activity (frequency, tension, and motility index) was calculated. The neural activation-mediated colonic muscle strip contraction was induced by electric field stimulation (EFS; 20 V, 2–64 Hz, 10 s pulse for an interval of 5 min). Data were analyzed using a polygraph (LabChart software 8.0).

### Western blot

Total protein in the colon tissue in each group was extracted using radio immunoprecipitation assay lysis buffer (Servicebio, G2002) with protease inhibitor (MedChemExpress, HY-B0496). The mixture was centrifuged at 12,000 g for 15 min, and the supernatants were collected and quantified using bicinchoninic acid method. An equal amount of protein samples was separated by 10% sodium dodecyl sulfate–polyacrylamide gel electro-phoresis and then transferred to polyvinylidene fluoride membrane. After blocking in 5% bovine serum albumin at 37 ℃ for 1 h, the membranes were incubated with the primary antibody (Additional file 2: Table S1) overnight at 4 °C. After rewarming and washing three times, the membranes were incubated with horseradish peroxidase-conjugated goat anti-mouse or goat anti-rabbit secondary antibodies (Antgene Biotech) for 1 h at room temperature. Protein bands were visualized using the chemi-luminescence imaging system (UVP, USA).

### Immunofluorescence

The Paraffin section of colon tissue was dewaxed and hydrated, and antigen retrieval was performed using sodium citrate. Fresh colon tissue was placed and washed in ice-cold Krebs buffer. The mucosa and submucosa were removed to expose the myenteric plexus, which was then fixed in 4% paraformaldehyde for 12 min. For the primary ENPCs in a 24-well plate, the cells were fixed in 4% paraformaldehyde for 30 min at room temperature. These preparations were incubated with 10% donkey serum containing 0.3% Triton X-100 to block non-specific binding and permeabilize. Next, these preparations were incubated with specific primary antibodies (Additional file 2: Table S1) overnight at 4 °C. After washing with PBS, the preparations were incubated with secondary antibody at 37 ℃ away from light for 2 h and subsequently labeled with 4’,6-diamidino-2-phenylindole (Antgene Biotech) for 10 min. The stained preparations were observed under confocal microscope (Nikon, Tokyo, Japan).

### Statistical analysis

GraphPad Prism 8.0c (San Diego, CA, USA) and ImageJ 1.52 were used for statistical analyses. Data are expressed as mean ± standard error. T-tests were used to compare the differences between two groups, and differences between multiple groups were compared using one-way analysis of variance. A *p* value < 0.05 was considered statistically significant, and statistically significant differences were defined as *p < 0.05, **p < 0.01, ***p < 0.001, and ****p < 0.0001.

## Results

###  L-Fucose promoted the recovery of gastrointestinal motility in diabetic mice

**Fig. 1:**
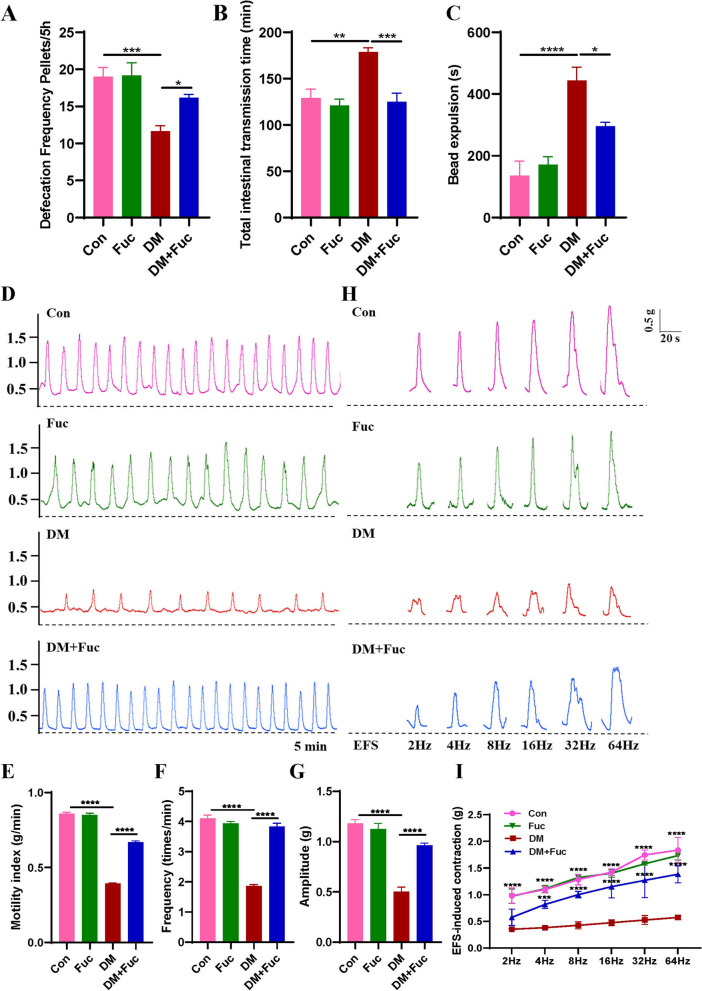
L-Fucose promoted the recovery of gastrointestinal motility in T1DM mice. **A** Defecation frequency (*n* = 6). **B** Total intestinal transmission time (*n* = 6). C Bead expulsion time (*n* = 6). **D** Representative basal curves of isometric tension of colonic muscle strip. Comparison of the motility index (**E**), frequency (**F**), and amplitude (**G**) of colonic muscle strip in each group (5 min) (*n* = 4). **H** Representative schematic diagram of colonic muscle strip contraction in response to EFS at different frequencies in each group (2–64 Hz). **I** Quantification of colonic muscle strip contraction in response to EFS in each group (n = 3). Con: the control mice; Fuc: control mice administrated with L-Fucose; DM: diabetic mice; DM + Fuc: diabetic mice administrated with L-Fucose; T1DM, type 1 diabetes mellitus; EFS, electrical field stimulation. Results were expressed as mean ± standard deviation. *p < 0.05, **p < 0.01, ***p < 0.001, ****p < 0.0001

To evaluate the effects of L-Fucose on gastrointestinal motility in diabetic mice, the mice were given L-Fucose for 28 consecutive days after eight weeks of injection of streptozotocin. Gastrointestinal motility was then measured both in vivo and ex vivo. Diabetic mice exhibited impaired gastrointestinal motility, indicated by a reduction in defecation frequency, elongation of total intestinal transmission time and bead expulsion time (all *p* < 0.01, Fig. 1A–C). Moreover, compared with the control group, the spontaneous contraction and EFS-induced contraction response of isolated colonic muscle strips in diabetic mice were weakened (*p* < 0.0001, Fig. 1D–I). However, gastrointestinal motility was recovered in diabetic mice after L-Fucose administration for 28 days, including increased defecation frequency (*p* < 0.05, Fig. 1A), reduced total intestinal transmission time (*p* < 0.001, Fig. 1B) and bead expulsion time (*p* < 0.05, Fig. 1C), as well as restored spontaneous contractility (*p* < 0.0001, Fig. 1D–G) and EFS-induced contraction response (*p* < 0.001, Fig. 1H–I) in isolated colonic muscle strips. We also evaluated the effects of L-Fucose on normal mice, and results showed no significant difference in gastrointestinal motility between the control group and the Fuc group throughout the study period (Fig. 1 and Additional file 1: Fig. S1B–D).

In addition, we also assessed the short-term effect of L-Fucose on gastrointestinal motility of diabetic mice. After oral administration of L-Fucose for 14 days, an acceleration of total intestinal transmission time was observed in diabetic mice (*p* < 0.05), whereas no significant differences were noted in terms of defecation frequency and bead expulsion time (Additional file 1: Fig. S1B–D).

### L-Fucose promoted the recovery of ENS by enhancing ENPCs-derived neurogenesis and gliogenesis in diabetic mice

**Fig. 2:**
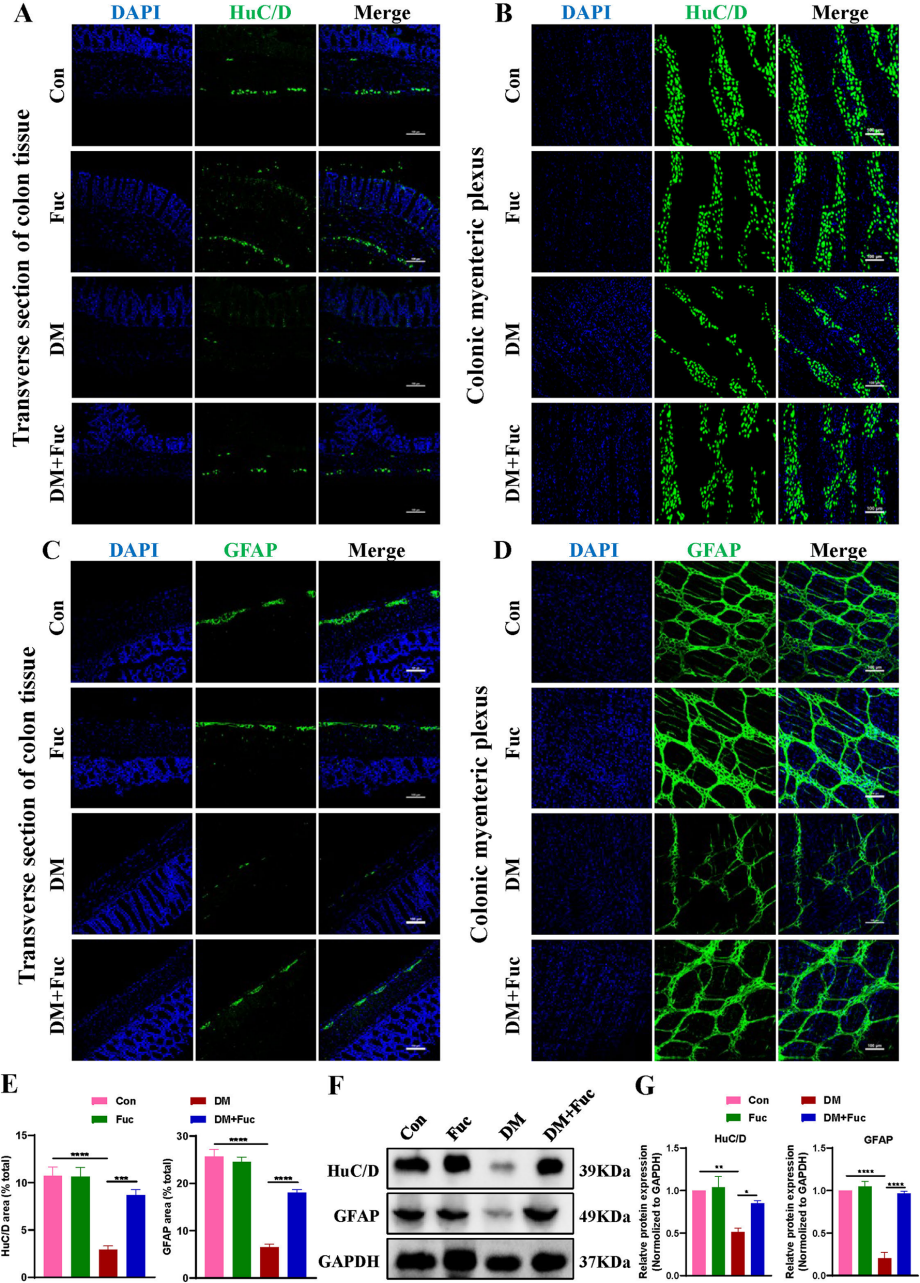
L-Fucose restored the colonic ENS structure in T1DM mice. **A** Representative immunofluorescence confocal laser images in transverse sections of colon tissue sections of HuC/D (green) in each group; the nuclei (blue). **B** Representative immunofluorescence confocal laser images in colonic myenteric plexus of HuC/D (green) in each group; the nuclei (blue). **C** Representative immunofluorescence confocal laser images in transverse sections of colon tissue of GFAP (green) in each group; the nuclei (blue). **D** Representative immunofluorescence confocal laser images in colonic myenteric plexus of GFAP (green) in each group; the nuclei (blue). **E** The statistics results of HuC/D and GFAP positive staining ratio in the colon myenteric plexus (*n* = 4). **F** Western blot analysis of HuC/D and GFAP proteins in the colon tissues. **G** Densitometric analysis of HuC/D and GFAP proteins in the colon tissues (*n* = 3). Con: the control mice; Fuc: control mice administrated with L-Fucose; DM: diabetic mice; DM + Fuc: diabetic mice administrated with L-Fucose; ENS, enteric nervous system; T1DM, type 1 diabetes mellitus. Results were expressed as mean ± standard deviation. *p < 0.05, **p < 0.01, ***p < 0.001, ****p < 0.0001

After observing alterations in gastrointestinal motility following L-Fucose treatment, we sought to determine whether these were associated with neuroanatomical changes in the ENS. Diabetic mice showed a reduction in the number of HuC/D^+^ neurons and GFAP^+^ glial cells in the myenteric plexus, while treatment with L-Fucose restored the number of HuC/D^+^ neurons and GFAP^+^ glial cells (*p* < 0.001, Fig. 2A-E). In line with this, we found that L-Fucose significantly upregulated the expression of HuC/D and GFAP proteins in the colon of DM mice (*p* < 0.05, Fig. 2F–G).

**Fig. 3:**
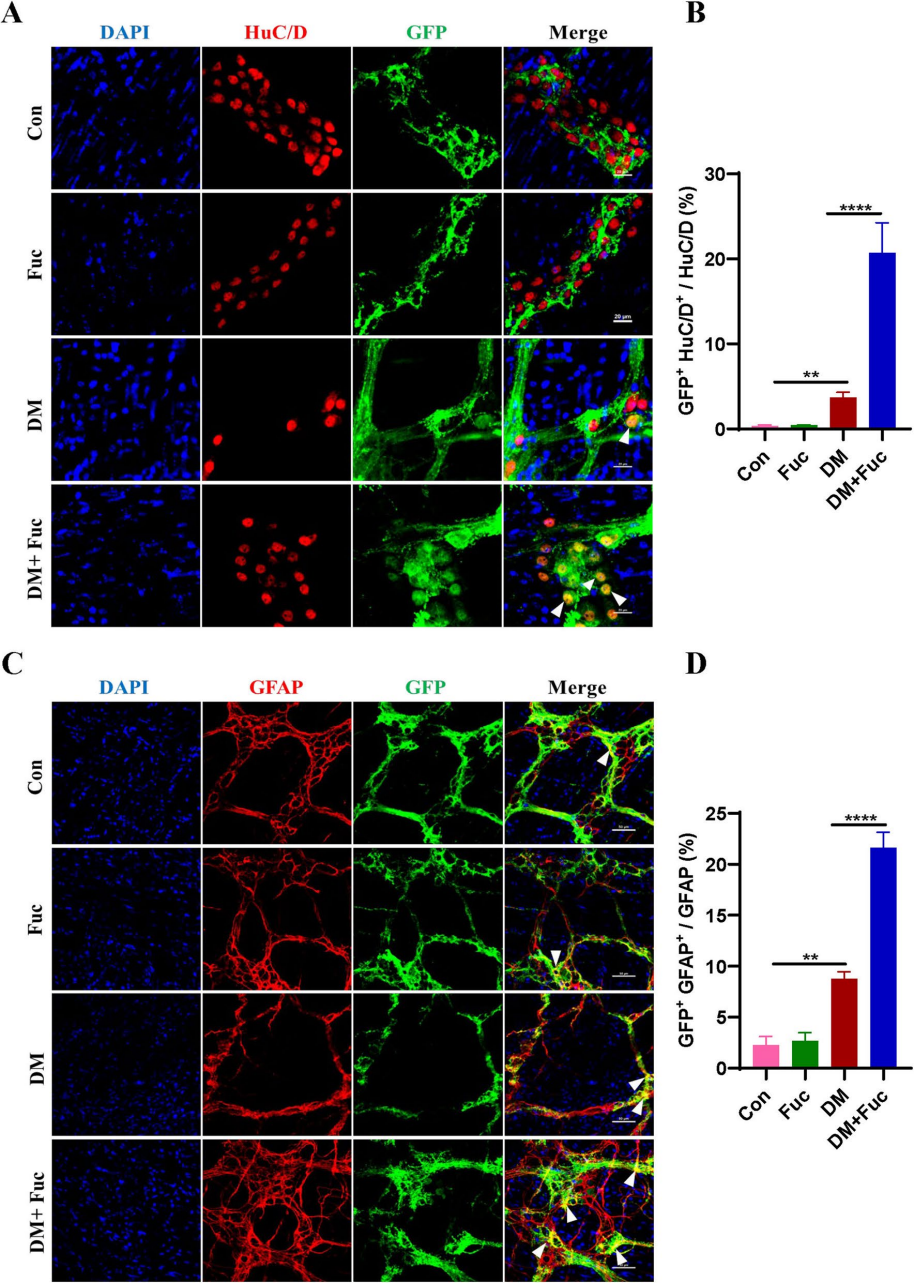
L-Fucose enhanced ENPCs-derived neurogenesis and gliogenesis in T1DM mice. **A** Representative immunofluorescence confocal laser images of neurons (HuC/D, red) co-expressed GFP (green) in colonic myenteric plexus in each group; the nuclei (blue). Arrows represent double-labeled HuC/D^+^ GFP^+^ neurons. **B** The proportion of HuC/D^+^ GFP^+^ neurons (newly formed neurons from ENPCs) in the colonic myenteric plexus in each group (*n* = 4). **C** Representative immunofluorescence confocal laser images of glial cells (GFAP, red) co-expressed GFP (green) in colonic myenteric plexus in each group; the nuclei (blue). Arrows represent double-labeled GFAP^+^ GFP^+^ glial cells. **D** The proportion of GFP^+^ GFAP^+^ glial cells (newly formed glial cells from ENPCs) in the colonic myenteric plexus in each group (*n* = 4). Con: the control mice; Fuc: control mice administrated with L-Fucose; DM: diabetic mice; DM + Fuc: diabetic mice administrated with L-Fucose; ENPCs, enteric neural precursor cells; T1DM, type 1 diabetes mellitus. Results were expressed as mean ± standard deviation. **p < 0.01, ****p < 0.0001

We next investigated the effects of L-Fucose on ENPCs within the colonic myenteric plexus. Nestin and Ngfr are the transcription factors known as the marker of ENPCs [9, 25]. Thus, we performed cell fate study using genetic lineage-tracing mice (Nestin-creER^T2^ × Ngfr-DreER^T2^: DTRGFP), in which cells derived from Nestin^+^/Ngfr^+^ cells would express GFP. In the control and Fuc groups, GFP is rarely co-expressed in enteric neurons and glial cells, whereas the percentage of GFP^+^ HuC/D^+^ double-labeled neurons in HuC/D^+^ neurons increased from 0.37% in the control group to 3.70% in the DM group (*p* < 0.01, Fig. 3A–B), and the percentage of GFP^+^ GFAP^+^ double-labeled glial cells in GFAP^+^ glial cells increased from 2.28% to 8.77% (*p* < 0.01, Fig. 3C–D). Notably, we observed significant increases in the proportion of GFP^+^ HuC/D^+^ double-positive neurons (*p* < 0.0001, Fig. 3A–B) and GFP^+^ GFAP^+^ double-positive glial cells (*p* < 0.0001, Fig. 3C–D) in the DM + Fuc group compared to the DM group.

Overall, our results suggest that ENPCs initiate compensatory self-repair in response to ENS damage, and L-Fucose can significantly enhance ENPCs-derived neurogenesis and gliogenesis, thereby repairing diabetes-induced dysfunctions in gastrointestinal motility and ENS.

### L-Fucose improved the proliferation and differentiation of injured ENPCs in vitro

**Fig. 4:**
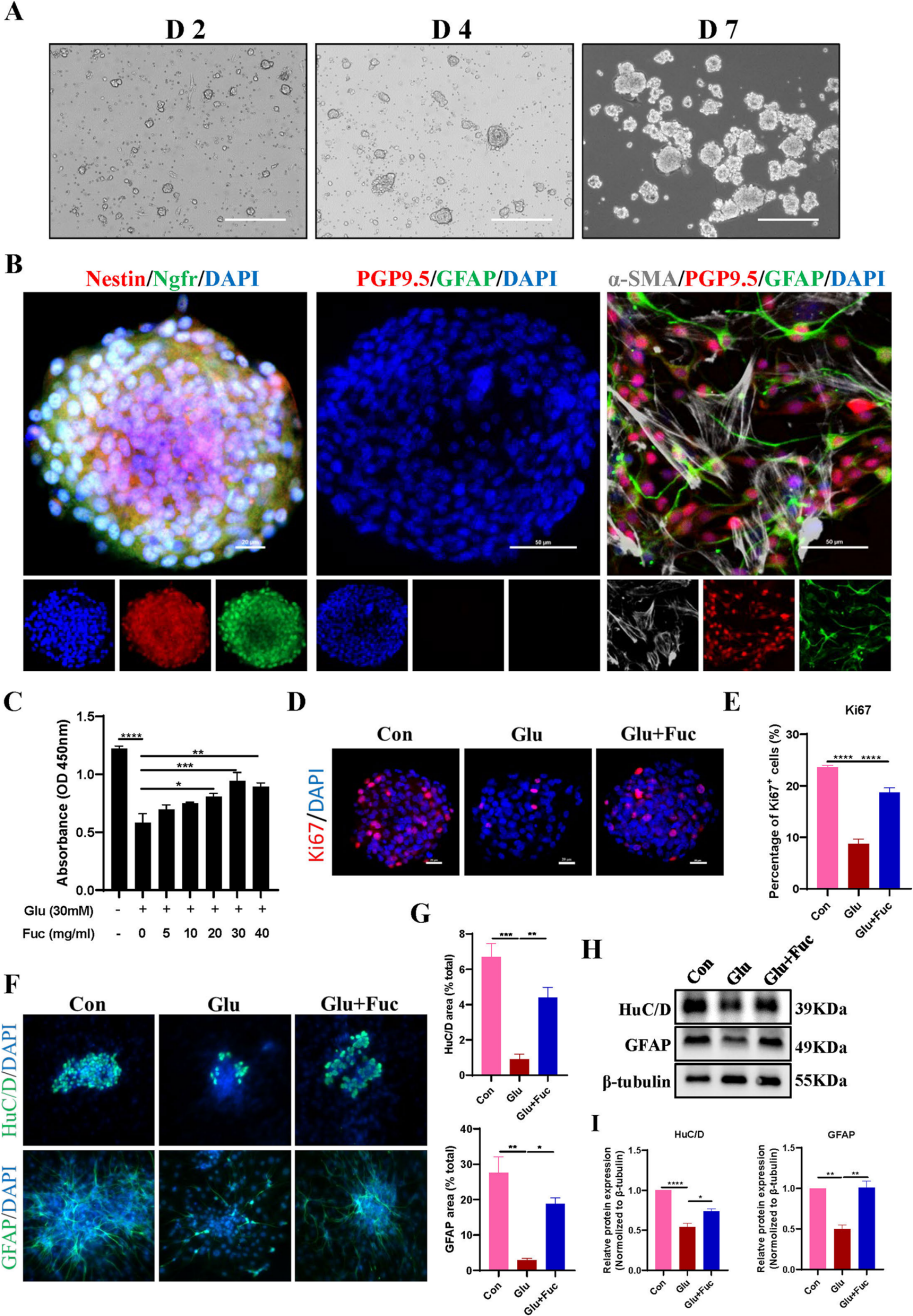
L-Fucose improved the proliferation and differentiation abilities of injured ENPCs in vitro. **A** Cell morphology of primary ENPCs at days 2, 4, and 7. **B** Immunofluorescence staining showed cells within neurospheres co-expressed Ngfr (green) and Nestin (red), but immuno-negative for glial cell marker GFAP (green) or neuronal marker PGP9.5 (red). By inducing differentiation, neurons (PGP9.5, red), glial cells (GFAP, green), and smooth muscle cells (α-SMA, gray) were derived from neurospheres; the nuclei (blue). **C** Cell viability of ENPCs treated with different concentrations of L-Fucose for 72 h was determined by CCK8 assay. **D** Ki67 staining of ENPCs in each group. **E** The positive rate of Ki67 was analyzed in the statistical chart. **F** Representative immunofluorescence images of ENPCs-derived neurons (HuC/D, green) or glial cells (GFAP, green) in each group; the nuclei (blue). **G** The positive rate of HuC/D and GFAP were analyzed in the statistical chart. **H** Western blot analysis of HuC/D and GFAP proteins in each group. **I** Densitometric analysis of HuC/D and GFAP proteins in each group. Con: Primary ENPCs cultures; Glu: ENPCs treated with 30 mM glucose; Fuc: ENPCs treated with 30 mM glucose and 30 mg/ml L-Fucose. ENPCs, enteric neural precursor cells. Results were expressed as mean ± standard deviation. *p < 0.05, ** *p* < 0.01, ***p < 0.001, ****p < 0.0001

Firstly, we characterized primary ENPCs in vitro, which were isolated from dissected intestines of embryonic mice (E15-E18). Neurospheres were formed after one week of culture (Fig. 4A), and immunostaining (Fig. 4B) showed that the cells within the neurospheres were positive for the ENPCs markers (Nestin and Ngfr), while negative for neuronal marker (PGP9.5) and glial cell marker (GFAP). After inducing differentiation for seven days, these cells were positive immunostaining for neuronal, glial, or smooth muscle cell (a-smooth muscle actin) markers. Collectively, these results indicate that we were able to isolate ENPCs in vitro, which could be used for subsequent studies.

To evaluate the effects of the high-glucose and L-Fucose on the proliferation and differentiation of ENPCs, primary ENPCs were maintained in culture medium containing 5- or -30 mM glucose, and treated with varying concentrations of L-Fucose.

The results of CCK8 assay (*p* < 0.0001, Fig. 4C) and Ki67 immunostaining (*p* < 0.0001, Fig. 4D–E) showed that high glucose significantly inhibited the proliferation of ENPCs, while treatment with L-Fucose demonstrated a dose-dependent improvement (5–40 mg/mL) in the proliferation of ENPCs damaged by high glucose, with the most optimal effect observed at 30 mg/mL (*p* < 0.001, Fig. 4C). Based on these findings, 30 mg/mL L-Fucose was selected for further experiments. Then, we assessed the impact of L-Fucose on the differentiation of ENPCs into neurons and glial cells. Immunofluorescence results showed that the proportion of ENPCs differentiated into neurons and glial cells decreased significantly under high glucose condition, but the abnormal differentiation induced by high glucose could be abolished by L-Fucose supplementation (HuC/D^+^ neuron: Control: 6.70 ± 1.31%; Glu: 0.91 ± 0.57%; and Glu + Fuc: 4.40 ± 1.00%, *p* < 0.01; GFAP^+^ glial cell: Control: 31.72 ± 4.10%; Glu: 2.90 ± 0.88%; and Glu + Fuc: 18.86 ± 2.86%, *p* < 0.05; Fig. 4F–G). Similarly, western blot analysis showed that the protein expressions of HuC/D and GFAP were downregulated under high glucose condition, while it was upregulated after L-Fucose supplementation (HuC/D: *p* < 0.05; GFAP: *p* < 0.01; Fig. 4H–I). In summary, our in vitro results suggest that L-Fucose supplementation can improve the proliferation and differentiation of injured ENPCs.

### L-Fucose promoted ENS regeneration through the SMAD2 signaling pathway, rather than the PI3K/AKT or ERK1/2 signaling pathways

**Fig. 5:**
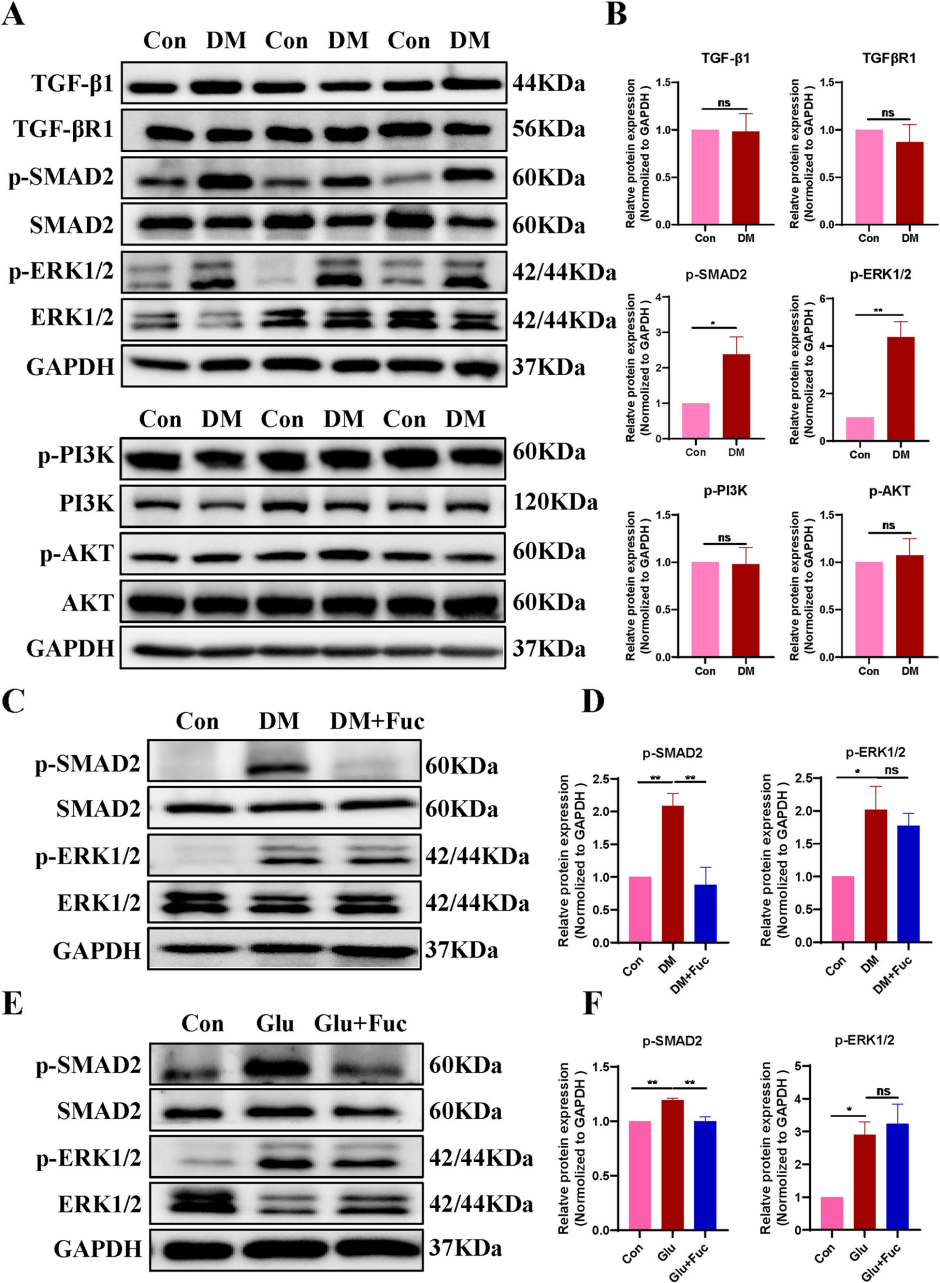
L-Fucose promoted ENS regeneration through inhibiting SMAD2 signaling pathway, but not PI3K/AKT or ERK1/2 signaling pathway. **A** Western blot analysis of TGF-β1 signaling pathway proteins, including SMAD2, p-SMAD2, ERK1/2, p-ERK1/2, TGF-β1, TGF-βR1, PI3K, p-PI3K, AKT and p-AKT in the colon tissues. **B** Densitometric analysis of TGF-β1 signaling pathway proteins in the colon tissues in each group (*n* = 3 to 4). **C** Western blot analysis of SMAD2 and ERK1/2 signaling pathways in the colon tissues. **D** Densitometric analysis of SMAD2 and ERK1/2 signaling pathways in the colon tissues in each group (*n* = 3 to 4). **E** Western blot analysis of SMAD2 and ERK1/2 signaling pathways in primary ENPCs cultures. **F** Densitometric analysis of SMAD2 and ERK1/2 signaling pathways in primary ENPCs cultures in each group (*n* = 3). Con: the control mice or primary ENPCs cultures; DM: diabetic mice; DM + Fuc: diabetic mice administrated with L-Fucose; Glu: ENPCs treated with 30 mM glucose; Fuc: ENPCs treated with 30 mM glucose and 30 mg/ml L-Fucose. ENPCs, enteric neural precursor cells. Results were expressed as mean ± standard deviation. *p < 0.05, **p < 0.01, ns, *p* > 0.05

**Fig. 6:**
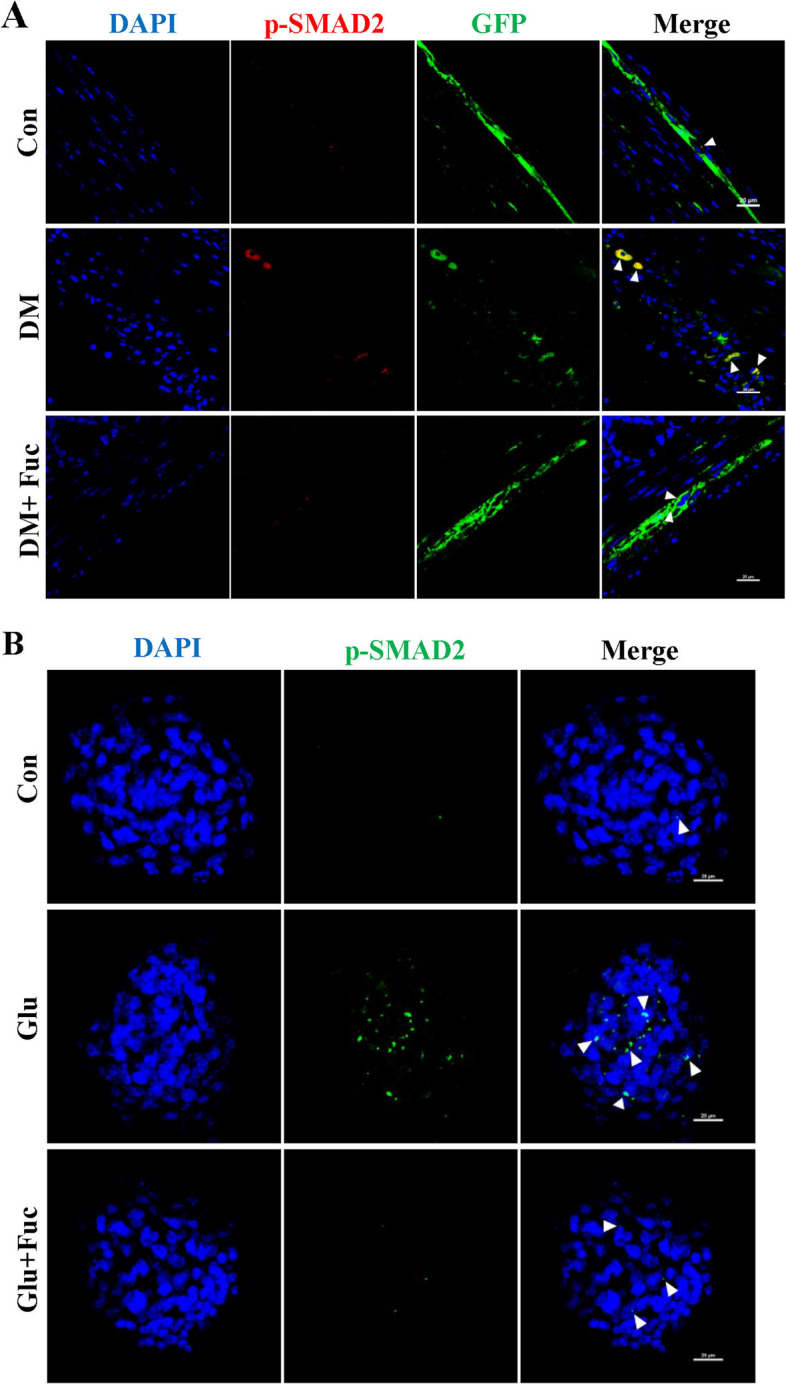
L-Fucose promoted ENPCs differentiation through inhibiting SMAD2 signaling pathway. **A** Immunofluorescence confocal laser images of GFP-labeled ENPCs (green) and p-SMAD2 (red) in each group in transverse sections of colon tissue; the nuclei (blue). Arrows represent double-labeled p-SMAD2^+^ GFP^+^ ENPCs. **B** Immunofluorescence staining of p-SMAD2 in ENPCs treated with glucose and L-Fucose. Arrows represent p-SMAD2^+^ expression. Con: the control mice or primary ENPCs cultures; DM: diabetic mice; DM + Fuc: diabetic mice administrated with L-Fucose; Glu: ENPCs treated with 30 mM glucose; Fuc: ENPCs treated with 30 mM glucose and 30 mg/ml L-Fucose. ENPCs, enteric neural precursor cells

As demonstrated above, L-Fucose could enhance ENPCs-derived neurogenesis and gliogenesis, thereby promoting the recovery of ENS and gastrointestinal motility in diabetic mice; therefore, we further explored the potential mechanisms. Given the pivotal involvement of the TGF-β signaling pathway in neurogenesis, we sought to determine whether L-Fucose functions by mediating the TGF-β signaling pathway of ENPCs. Compared with the control group, the phosphorylation levels of SMAD2 and ERK1/2 were significantly enhanced in DM mice (*p* < 0.05, Fig. 5A–B), while the protein levels of TGF-β1, TGF-β receptor I (TGF-βR1), and the phosphorylation levels of PI3K and AKT had no significant changes (*p* > 0.05, Fig. 5A–B). We then focused on the effects of L-Fucose on ERK1/2 and SMAD2 signaling pathways. The results showed that L-Fucose significantly inhibited the phosphorylation level of SMAD2 in DM mice (*p* < 0.01, Fig. 5C–D), but not that of ERK1/2 (*p* > 0.05, Fig. 5C–D). In addition, L-Fucose treatment did not affect the protein expression level of SMAD2 signaling in control mice (*p* > 0.05, Additional file 1: Fig. S1E-F). A similar tendency was observed in primary ENPCs (p-SMAD2, *p* < 0.01; p-ERK1/2, *p* > 0.05; Fig. 5E–F). In addition, immunostaining data also confirmed an elevated level of p-SMAD2 in the injured ENPCs was observed both in vivo and in vitro, and this tendency was reversed after L-Fucose treatment (Fig. 6).

## Discussion

The increased prevalence of DM is associated with high incidence of complications and has caused an enormous socioeconomic impact. As reported, up to 75% of individuals with diabetes are complicated by gastrointestinal motility dysfunction over time [26, 27]. The ENS plays a critical role in maintaining gastrointestinal motility, and researches suggests that ENPCs are a promising therapeutic target for diseases related to ENS damage. In this study, we found that diabetes induced loss of enteric neurons and glial cells, accompanied by compensatory increases in ENPCs-derived neurogenesis and gliogenesis. L-Fucose treatment restored the number of enteric neurons and glial cells by markedly enhancing ENPCs-derived neurogenesis and gliogenesis, thus improving gastrointestinal motility in T1DM mice.

Growing evidence has demonstrated the essential role of ENPCs-derived neurogenesis in the maintenance of ENS-dependent functions. Pasricha et al. revealed that ENPCs govern neurogenesis to maintain a dynamic balance of enteric neuron in the healthy adult small intestine [9]. Intestinal microbiota modulated the structure and function of the ENS by affecting ENPCs-derived neurogenesis [10, 28]. In addition, our previous study showed that mesenchymal stem cells transplantation promoted differentiation of ENPCs to restore gastric motility in mice with pylorus denervation [25]. In this study, we employed ENPCs transgenic reporter mice (Nestin-creER^T2^ × Ngfr-DreER^T2^: DTRGFP), and observed that ENPCs exhibited a reversible state of dormancy in the adult colon, characterized by a low rate of neurogenesis and gliogenesis under physical condition, while damage induced by diabetes evoked the neurogenic response of ENPCs. Vicentini et al. found that gut bacteria depletion led to an indiscriminate loss of enteric neurons, triggering enteric neurogenesis in the myenteric plexus [29]. Moreover, colitis induced enteric neurogenesis through a 5-HT4-dependent mechanism [30]. These findings indicated that injury or inflammation can activate the neurogenic capacity of ENPCs. However, the increase in the number of neurons and glial cells caused by ENPCs differentiation was significantly lower than the loss of enteric neurons and glial cells induced by diabetes, which resulted in a decrease in the absolute number of enteric neurons and glial cells in diabetic mice, further suggesting the need for modulators that can enhance the neurogenic potential of ENPCs.

L-Fucose has great potential in the treatment of neurodegenerative diseases [31, 32]. In the current study, we found that L-Fucose exerted significant effects on gastrointestinal motility recovery and ENS regeneration in diabetic mice. Moreover, there was a notable increase in the proportion of ENPCs-derived neurons and glial cells following L-Fucose treatment, both in vivo and in vitro. Based on our results, the increase of neurogenesis and gliogenesis derived from ENPCs should be considered as the foundational for L-Fucose-induced ENS regeneration in T1DM mice. Therefore, as a non-toxic dietary sugar with a safety precedent experimental therapy for children with leukocyte adhesion deficiency type II [33], L-Fucose appears to be a potentially safe and tolerable therapeutic agent for the treatment of ENS-related disorders. Further investigation is warranted to explore the therapeutic effects of L-Fucose on congenital megacolon, type 2 diabetes and other gastrointestinal motility disorders [34, 35].

Recent studies highlight the role of the TGF-β signaling pathway as a master regulator of stem cell differentiation [36, 37]. Moreover, studies support that L-Fucose and its analogs have extensive cross-talk with TGF-β signaling in various diseases [38–41]. Zhang et al. demonstrated that fucoidan ameliorates pulmonary fibrosis by inhibiting both the PI3K/AKT and TGF-β/SMAD signaling pathways [42]. Similarly, Ramil et al. found that fucoidan exerts its protective effects in an in vitro model of synovial fibrosis by blocking the TGF-β/SMAD2 pathway [43]. Correspondingly, here, we observed a significant increase in the phosphorylation levels of ERK1/2 and SMAD2 in the injured ENPCs both in vitro and in vivo. L-Fucose treatment significantly reduced the phosphorylation of SMAD2 in the injured ENPCs, while no significant change was found on the phosphorylation level of ERK1/2. Furthermore, our study did not observe significant differences in the expression levels of TGF-β1 and TGF-βR1. TGF-β signaling can be modulated by post-translational modifications. So far, sumoylation, glycosylation, and fucosylation have been shown to post-translationally modify the TGF-β1 and TGF-βR1 proteins [44–46]. In addition, latent TGF-β1 released from the extracellular matrix can activate SMAD2 signaling independently of the expression levels of TGF-β1 and TGF-βR1 [47, 48]. Taken together, these results suggest that L-Fucose promotes ENS regeneration through inhibiting SMAD2 signaling in ENPCs in T1DM mice.

In conclusions, the present study indicates that ENPCs presents a low rate of neurogenesis and gliogenesis under physical condition, whereas activates and differentiates into enteric neurons and glial cells upon injury. L-Fucose treatment significantly enhances ENPCs-derived neurogenesis and gliogenesis through suppressing SMAD2 phosphorylation, thus promoting ENS regeneration and gastrointestinal motility recovery in T1DM mice. The elucidation of the alterations of ENPCs within the ENS is expected to advance our understanding of ENS-related disorders and facilitate the future application of L-Fucose as a therapeutic agent.

### Supplementary Information

**Additional file 1: Figure S1.** (A) Immunostaining showed the co-expressed of GFP (green), Nestin (red), and Ngfr (purple) in colonic myenteric plexus in Nestin-creERT2 × Ngfr-DreERT2: DTRGFP triple transgenic mice. (B-D) The effects of L-Fucose on gastrointestinal motility in diabetic mice by oral gavage for continuous 14 days (n = 5), including defecation frequency (B), The total intestinal transmission time (C), and bead expulsion time (D). (E) The expression level of SMAD2 signaling in control and Fuc groups. (F) Densitometric analysis of SMAD2 signaling in control and Fuc groups. Con: the control mice; Fuc: control mice administrated with L-Fucose; DM: diabetic mice; DM + Fuc: diabetic mice administrated with L-Fucose. Results were expressed as mean ± standard deviation. *p < 0.05, ****p < 0.0001, ns, *p* > 0.05

**Additional file 2:  Table S1. **The primary and secondary antibodies used for western blot analysis, and immunofluorescence staining

## Data Availability

All data analyzed during this study are included in this article.

## Abbreviations

CNS: Central nervous system
DM: Diabetes mellitus
DMEM/F12: Dulbecco’s Modified Eagle Medium/Nutrient Mixture F-12
EFS: Electric field stimulation
ENPCs: Enteric neural precursor cells
ENS: Enteric nervous system
ERK: Extracellular signal-regulated kinase
GFAP: Glial fibrillary acidic protein
GFP: Green fluorescent protein
NPCs: Neural precursor cells
PBS: Phosphate buffered saline
P/S: Penicillin/Streptomycin
T1DM: Type 1 diabetes mellitus
TAM: Tamoxifen
TGF-β: Transforming growth factor-β
TGF-β1R: TGF beta receptor I
