# Supplementary figures and images for: L-Fucose promotes enteric nervous system regeneration in type 1 diabetic mice by inhibiting SMAD2 signaling pathway in enteric neural precursor cells
Source: Cell Commun Signal. 2023 Oct 5;21:273. doi: 10.1186/s12964-023-01311-0 (PMC10552466; doi:10.1186/s12964-023-01311-0)

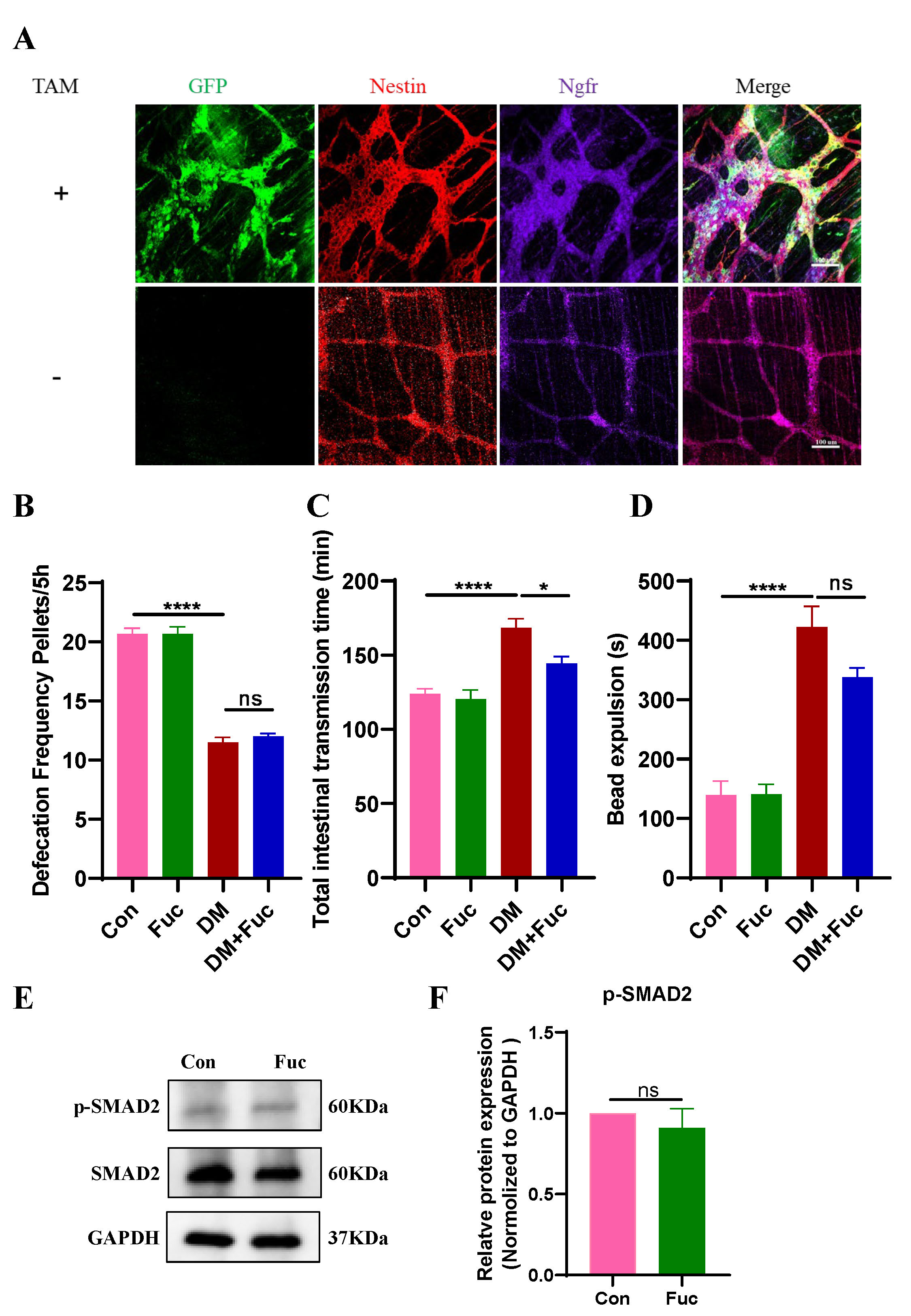

Supplement: Supplementary file 2 — Additional file 1: Figure S1. (A) Immunostaining showed the co-expressed of GFP (green), Nestin (red), and Ngfr (purple) in colonic myenteric plexus in Nestin-creERT2 × Ngfr-DreERT2: DTRGFP triple transgenic mice. (B-D) The effects of L-Fucose on gastrointestinal motility in diabetic mice by oral gavage for continuous 14 days (n = 5), including defecation frequency (B), The total intestinal transmission time (C), and bead expulsion time (D). (E) The expression level of SMAD2 signaling in control and Fuc groups. (F) Densitometric analysis of SMAD2 signaling in control and Fuc groups. Con: the control mice; Fuc: control mice administrated with L-Fucose; DM: diabetic mice; DM + Fuc: diabetic mice administrated with L-Fucose. Results were expressed as mean ± standard deviation. *p < 0.05, ****p < 0.0001, ns, p > 0.05 [file 12964_2023_1311_MOESM1_ESM.jpg]
