# Supplementary material for: L-Fucose promotes enteric nervous system regeneration in type 1 diabetic mice by inhibiting SMAD2 signaling pathway in enteric neural precursor cells
Source: Cell Commun Signal. 2023 Oct 5;21:273. doi: 10.1186/s12964-023-01311-0 (PMC10552466; doi:10.1186/s12964-023-01311-0)
Supplement: Supplementary file 3 — Additional file 2: Table S1. The primary and secondary antibodies used for western blot analysis, and immunofluorescence staining [file 12964_2023_1311_MOESM2_ESM.docx]

**Table S1. The primary and secondary antibodies used for western blot analysis, and immunofluorescence staining**

| **Primary antibody** | **Producer**  **(Catalog Number)** | **Concentration** |
| --- | --- | --- |
| Rabbit anti-HuC/D | Abcam (ab184267) | IF (1:500); WB (1:1000) |
| Rabbit anti-PGP9.5 | Abclonal (A19101) | IF (1:200) |
| Rabbit anti-GFAP | Abclonal (A0237) | IF (1:200); WB (1:1000) |
| Goat anti-GFP | Abcam (ab5450) | IF (1:1000); WB (1:5000) |
| Rabbit anti-Ki67 | Abcam (ab16667) | IF (1:200) |
| Rabbit anti-Nestin | Abclonal (A11861) | IF (1:200) |
| Mouse anti-Ngfr | R/D system (AF1157) | IF (1:200) |
| Mouse anti-α-SMA | Boster (BM0002) | IF (1:200) |
| Rabbit anti-p-SMAD2 (Ser465/467) | CST (3108) | IF (1:200); WB (1:1000) |
| Rabbit anti-SMAD2 | CST (5339) | WB (1:1000) |
| Rabbit anti-p-ERK (Thr202/Tyr204) | CST (4370) | WB (1:1000) |
| Rabbit anti-ERK | CST (4696) | WB (1:1000) |
| Rabbit anti-p-PI3K | CST (17366) | WB (1:1000) |
| Rabbit anti-PI3K | CST (4257) | WB (1:1000) |
| Rabbit anti-p-AKT | CST (4060) | WB (1:1000) |
| Rabbit anti-AKT | CST (4691) | WB (1:1000) |
| Rabbit anti-TGF-β1 | Abclonal (A2124) | WB (1:1000) |
| Rabbit anti-TGF-βR1 | Abclonal (A16983) | WB (1:1000) |
| Rabbit anti-GAPDH | Antgene (ANT325) | WB (1:1000) |
| **Secondary antibody** |  |  |
| HRP-labelled anti-rabbit antibody | AntGene | WB (1:3000) |
| HRP-labelled anti-goat antibody | AntGene | WB (1:3000) |
| Alexa Fluor 488 and donkey anti-goat IgG | AntGene | IF (1:200) |
| Alexa Fluor 488 and donkey anti-rabbit IgG | AntGene | IF (1:200) |
| Alexa Fluor 594 and donkey anti-rabbit IgG | AntGene | IF (1:200) |
| Alexa Fluor 594 and donkey anti-mouse IgG | AntGene | IF (1:200) |
